# Characterization and Transcriptomic Analysis of Sorghum EIN/EIL Family and Identification of Their Roles in Internode Maturation

**DOI:** 10.3390/plants13182615

**Published:** 2024-09-19

**Authors:** Min Tu, Yuqing Hua, Ti Shao, Siyu Zhang, Zihan Xiang, Manting Yu, Guoli Wang, Zhuang Li, Yun He, Lin Yang, Yin Li

**Affiliations:** 1 Hubei Technical Engineering Research Center for Chemical Utilization and Engineering Development of Agricultural and Byproduct Resources, School of Chemical and Environmental Engineering, Wuhan Polytechnic University, Wuhan 430023, China; 2 The Genetic Engineering International Cooperation Base of Chinese Ministry of Science and Technology, Key Laboratory of Molecular Biophysics of Chinese Ministry of Education, College of Life Science and Technology, Huazhong University of Science and Technology, Wuhan 430074, China; 3 School of Mathematics and Computer Science, Wuhan Polytechnic University, Wuhan 430023, China

**Keywords:** EIN/EIL family, gene expansion, RNA-seq analysis, internode maturation, co-expression analysis, sorghum, transcription factors

## Abstract

Ethylene-insensitive 3/Ethylene-insensitive3-like proteins (EIN3/EIL) represent a group of transcription factors critical for the ethylene signaling transduction that manipulate downstream ethylene-responsive genes, thereby regulating plant growth, development, and stress responses. However, the identification, evolution, and divergence of the EIL family remain to be studied in *Sorghum bicolor*. Here, we identified eight *SbEIL*s, which were expanded due to whole-genome-duplication (WGD) events. Characterization of the protein sequences and expression atlas demonstrates that the WGD-duplicated *SbEIL*s could become divergent due to the differential expression patterns, rather than domain and motif architectures. Comparative expression analysis was performed between the RNA-seq data sets of internodes from several sorghum cultivars to understand the potential roles of SbEIL members in internode elongation and maturation. Our results identified *SbEIL3* and *7* (the latter as a homolog of *OsEIL7/OsEIL1*) to be the highly expressed *SbEIL* genes in sorghum internodes and revealed a potential functional link between *SbEIL7* and internode maturation. The co-expression analysis and comparative expression analysis with ethylene-regulated gene sets found that *SbEIL7* was co-regulated with a set of ubiquitin-related protein degradation genes, suggesting possible involvement of SbEIL7 in protein degradation and processing during the post-anthesis stages. Altogether, our findings lay a foundation for future functional studies of ethylene signaling-mediated gene regulation and improvement of sorghum internode development.

## 1. Introduction

Ethylene is a gaseous phytohormone that affects various plant developmental processes, including seed germination, flower and leaf senescence, fruit ripening, root development, programmed cell death, responsiveness to biotic and abiotic stresses, etc. [1,2,3,4]. Ethylene signaling starts with receptors sensing ethylene in plants. The N-terminal region of CTR1 (Constitutive Triple Response 1) interacts with ethylene receptors, while the C-terminus is a Raf-like Ser/Thr protein kinase domain [5,6,7]. In the absence of ethylene, CTR1 inhibits downstream ethylene responses by phosphorylating the C-terminus of EIN2 (Ethylene-insensitive 2) [8]. On the contrary, the presence of ethylene inactivates the receptor and CTR1, reduces the phosphorylation of EIN2, resulting in its C-terminal fragment being cleaved and transferred into the nucleus, and finally activates the transcriptional cascade of EIN3/EIL1 (Ethylene-insensitive 3/Ethylene-insensitive3-like proteins) [9].

EIN3/EIL1 acts as the master transcription factors in the ethylene signaling pathway, manipulating downstream genes or regulators associated with ethylene responses, such as ERF1 (Ethylene Response Factor), which further regulate diverse aspects of plant growth, development, and stress responses [6,10,11,12,13,14].

*EIL1* belongs to a gene family of transcription factors designated as *EIN3/EIL* (abbreviated as the EIL family hereafter) that encodes proteins containing the EIN3 domain [15], of which the structures are mainly characterized by highly conserved N-terminal and less conservative C-terminal sequences, suggesting the various functions of *EIL1* homologs in different plant species [10]. In the model species, such as *Arabidopsis thaliana* and rice (*Oryza sativa*), EIL family members have been known to regulate not only the processes of plant growth and development but also various molecular responses to biotic and abiotic stresses [1,16,17].

As a multigenic family, functional studies of the EIL members were mainly concentrated in the dicot model Arabidopsis. Recently, the roles of EIL members have been gradually explored in monocot crops, especially rice. In rice, *OsEIL1* has higher expression levels than that of *OsEIL2*. Both *OsEIL1* and *2* are repressed with the ethylene treatment and are wound-inducible [18]. In the rice *maohuzi6* mutant (*MHZ6*), *MHZ6* encodes OsEIL1, with *OsEIL1* mutations leading to ethylene insensitivity mainly in roots, but *OsEIL2* mutations cause ethylene insensitivity mainly in coleoptiles [19]. OsEIL plays a major role in ethylene signaling with pleiotropic effects. For example, OsEIL is involved in the crosstalk between ethylene- and jasmonic acid-pathway responses to a piercing–sucking insect in rice [20]; OsEIL targets the promoter of *OsERF115* to control grain size and weight [21]; and OsEIL also participates in positive feedback regulation of ethylene signaling by auxin biosynthesis for root growth [22].

Genome-wide analysis of the *EIL* family in major staple crops (i.e., rice and maize) and functional characterization of several OsEILs serve as the foundation for reverse genetic studies and comparative genomic studies to bridge the ethylene-related knowledge between the major monocot model species and the other understudied species (like sorghum) [18,19,20,21,22,23,24]. The *EIL* family from both rice and maize consists of nine members [25,26], while 21 *EIL* members were identified in wheat [27]. According to phylogenetic analyses, *OsEIL1/2* (LOC_Os03g20780 and LOC_Os07g48630, respectively; the former also named as *OsEIL7* by Aluko et al.) [26], *TaEIL1*, and *ZmEIL1/9* are homologous to the master regulators in the ethylene signaling pathway *AtEIL1/EIN3*, indicating that these *EIL* homologs from crops may also play crucial roles in ethylene-mediated biological processes in crops.

Importantly, the evolution of Poaceae grass species (e.g., rice, maize, sorghum, and Brachypodium) has been extensively studied, and the whole-genome duplication events (e.g., τ-WGD and ρ-WGD) of the common ancestor of Poaceae have greatly shaped the genomic contents of the grass species [28,29,30,31]. The established evolutionary relationships between these grass genomes help understand and translate gene functions from the model species (rice, for instance) to the understudied relatives (e.g., sorghum). Indeed, analytical strategies have been established in the Triticeae species with similar concepts as integrative gene duplication and genome-wide analysis [32]. In the present study, to infer the evolution trajectory of *SbEIL*s and to lay a foundation for future functional studies about ethylene signaling-mediated gene regulation in sorghum, we performed genome-wide identification of *SbEIL*s and particularly analyzed the evolution between *SbEIL*s and their orthologs in maize and rice (*ZmEIL*s and *OsEIL*s), highlighting the WGD-driven expansion of the *SbEIL* family. Comprehensive expression analyses demonstrated that WGD-duplicated *SbEIL*s could become divergent due to differentiated expression patterns. Expression analyses focused on the internode tissues indicated that *SbEIL3* and *7* might have previously unknown roles during the process of internode elongation and maturation.

## 2. Results and Discussion

### 2.1. Ancient Whole-Genome Duplication Events Drive the Expansion of the EIL Family in Rice, Maize, and Sorghum

**Figure 1:**
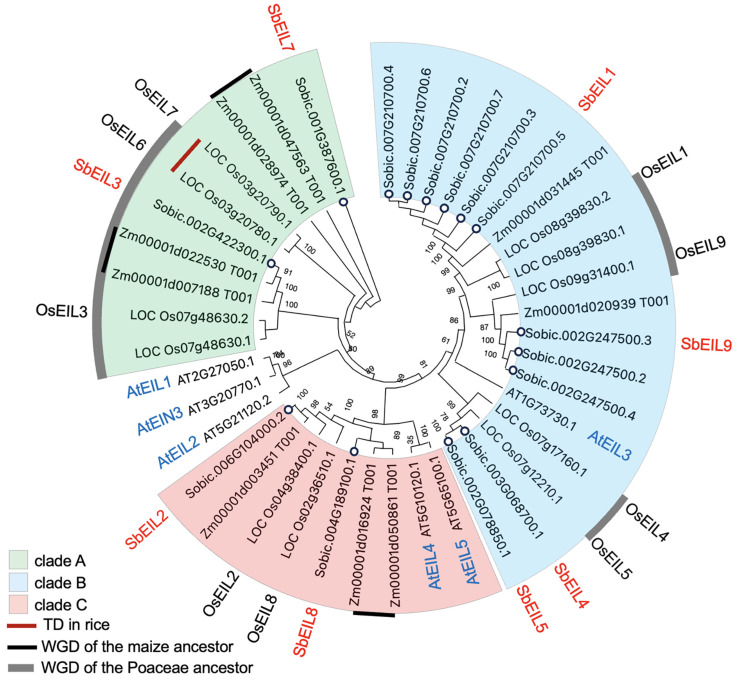
Phylogenetic analysis of the SbEIL family. The protein sequences of SbEILs, OsEILs, ZmEILs, and AtEILs were used in the phylogenetic analysis, with the EIL protein sequences aligned with the MUSCLE method and the tree constructed with the maximum-likelihood method by using the MEGA-X software. A total of 1000 bootstrap replicates were applied, with the supporting values for each branch shown in the tree. The nomenclature of AtEILs is according to a previous study and labeled in blue font in the figure, while the OsEILs and SbEILs are named following Aluko et al. and labeled with black and red fonts, respectively [26]. EILs from the monocot species (i.e., rice, maize, and sorghum) are phylogenetically divided into three clades (A, B, and C, shown with the background color green, blue, and red, respectively). Red bars indicate tandem duplicated OsEIL genes, while the black bars indicate the whole-genome-duplicated ZmEILs during the paleo-tetraploidization process of the maize genome [35]. Gray arcs indicate the OsEILs generated by the ancient whole-genome duplication events of the Poaceae species [28,29]. SbEILs are highlighted with white dots. Predicted SbEIL proteins from multiple encoding transcripts were used in the phylogenetic analysis, whereas the longest OsEILs and ZmEILs produced from the same gene were used.

We combined the BLAST-based and protein domain search-based methods to identify *SbEIL* genes in sorghum. A total of eight *SbEIL*s were identified. According to the annotated transcripts and predicted protein sequences, these *SbEIL* genes putatively encode 16 proteins. To understand their orthologous relationships with *OsEIL*s and *AtEIL*s, we performed phylogenetic analysis with the protein sequences of the identified SbEILs, together with nine ZmEILs, nine OsEILs, and six AtEILs [25,27,33]. The monocot EIL proteins fall into three phylogenetic clades (i.e., clades A, B, and C) (Figure 1). Possibly owing to the far evolutionary relationships between the dicot model species *Arabidopsis thaliana* and the monocot species, AtEIL1, AtEIL2, and AtEIN3 were not grouped together with the clade A EILs but represent the AtEILs closest to clade A. Considering the critical roles of AtEIL1/2 and AtEIN3 in the ethylene signal pathway, the clade A EILs may likely be the homologs both evolutionarily and functionally [6,10]. Notably, the OsEIL nomenclature in our work was according to Aluke et al. [26]. Even though they were identified in the previous study, the evolutionary information and expression profiles of *SbEILs* have not been characterized in detail [26]. The Poaceae grass species feature unique WGD events, including the one that happened in the Poaceae ancestor and the paleo-tetraploidization event specific to modern maize [28,29,30,31]. Taking advantage of this Poaceae WGD information, we found that, generally, the monocot *EIL* family expands due to WGD events. In the rice genome, three pairs of WGD-derived *OsEIL*s were identified, namely *OsEIL1/9* (LOC_Os08g39830 and LOC_Os09g31400), *OsEIL4/5* (LOC_Os07g17160 and LOC_Os07g12210), and *OsEIL3/7* (LOC_Os07g48630 and LOC_Os03g20790), while *OsEIL6* and *7* were produced probably by tandem duplication. Based on the reciprocal BLAST search between rice, maize, and sorghum and the established syntenic orthologous relationships between sorghum and maize, we determined that the WGD-duplicated pairs (i.e., *OsEIL1/9*, *OsEIL4/5*, and *OsEIL3/7*) were maintained in the sorghum BTx623 genome, leading to the sorghum WGD-duplicated *EIL* pairs, *SbEIL1/9*, *SbEIL4/5*, and *SbEIL3/7* [34,35]. The tandemly duplicated *OsEIL6* and *7* appear to be rice-specific. The paleo-tetraploidization event of the maize genome contributed to the expansion of *ZmEIL*s, as Zm00001d047563 and Zm00001d028974, Zm00001d022530 and Zm00001d007188, and Zm00001d016924 and Zm00001d050861 are specifically duplicated in the maize genome according to the previous genomic studies [30,31,35]. In short, phylogenetic and evolutionary analyses revealed that ancient whole-genome duplication events drove the expansion of the *EIL* family in rice, maize, and sorghum.

### 2.2. Distinct Expression Patterns Could Contribute to the Divergence of Duplicated SbEILs

We sought to address whether the WGD-duplicated *SbEIL*s have redundant or divergent functions. Duplicated genes could evolve to have different architectures of domains and/or motifs, or adopt distinct expression profiles, allowing for functional divergence. The SbEIL proteins vary in length, ranging from 322 amino acids (AAs) to 643 AAs, with the molecular weight ranging from 33.7 kDa to 71.8 kDa (Table 1). The SbEILs were acidic proteins with their isoelectric points (pI) varying from 4.94 to 6.05. Based on the prediction, SbEIL1 could be stable, while the remaining SbEILs might be unstable. The aliphatic index of the SbEILs is predicted to range between 59.9 and 80.3. The aliphatic index (AI) is directly related to the mole fraction of Ala, Ile, Leu, and Val in the protein [36] and is a positive factor for the thermo-stability of proteins [37]. SbEIL proteins are predicted to be localized in the nucleus, consistent with their roles as transcription factors.

**Figure 2:**
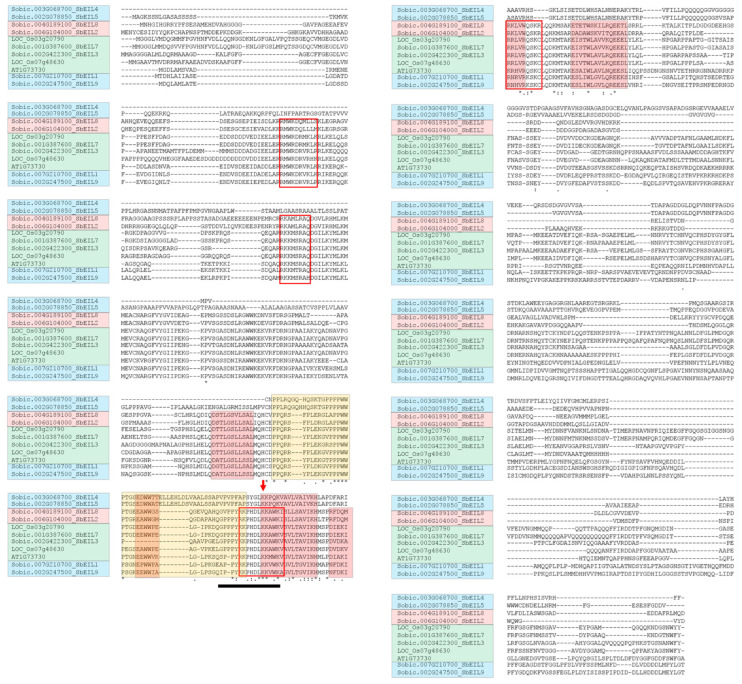
Alignment of the protein sequences identifies the conserved domains in the EIL proteins. Full-length protein sequences of SbEILs and representative EILs (AtEILs and OsEIL7) were aligned with the MUSCLE method, with their phylogenetic clades indicated with the same background colors in Figure 1. In the N-terminal DNA-binding domain, the five predicted α-helix regions are highlighted with a red background, while the proline-rich region is indicated with a yellow background. The red arrowhead indicates the Arabidopsis *ein3-3* mutational site, which is very conserved between the proteins [38]. The red boxes indicate the four small clusters of basic amino acids (known as the basic domains, BD I, BD II, BD III, and BD IV). * indicates the amino acid residues that are conserved among the EIL proteins used for the alignment. The black underline indicates the putative nuclear localization signal (amino acid sequence “GPPPYKKPHDLKKAWK”) [38,39,42].

**Figure 3:**
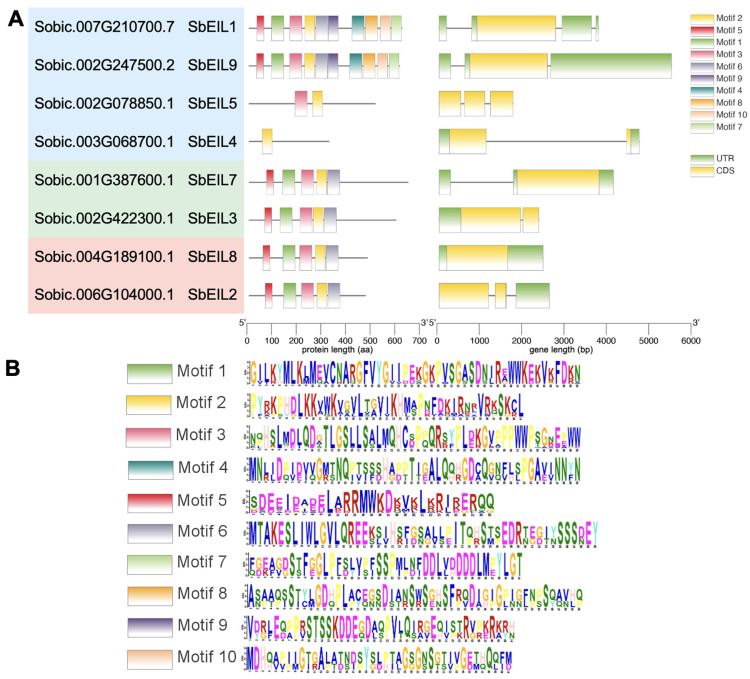
The conserved motif and gene architecture of the EIL proteins. (**A**) The MEME-predicted conserved motifs of SbEILs and the exon–intron architectures of the corresponding encoding genes [43]. (**B**) Motif logo of the EIL family. Ten motifs were predicted with the MEME software.

Alignment of the SbEIL protein sequences with representative EILs (AtEIL3 and OsEIL7) found that the N-terminal regions of most SbEILs are conserved with AtEIL3 and OsEIL7 (Figure 2). In the N-terminal region of EIL, five conserved α-helices were identified in SbEILs except for SbEIL4 and SbEIL5. Structural studies and predictions have determined that these five α-helices form a V-shape DNA-binding domain critical for the function of the EIL transcription factor. In addition, the proline-rich region of EIL proteins contributes to the main-chain/side-chain interactions and the packing of α-helices 2 and 3 [38,39]. Indeed, the proline-rich region was detected in all of the SbEILs, and these proline-rich regions contain α-helix 2 and the adjacent loops. In addition, we also observed four basic amino acid domains (highlighted in red boxes in Figure 2) that have been considered as the conserved domains important for EIL proteins’ functions [38]. In order to describe the structural diversity of SbEIL-encoding genes and the proteins, we analyzed the exon–intron structure of *SbEIL* genes and predicted the potentially conserved motifs with MEME (Figure 3). The MEME-predicted motifs 1, 2, 3, 5, and 6 were conserved among all SbEILs except for SbEILs 4 and 5. This finding is in agreement with the sequence alignment results that SbEIL4 and 5 have a distinct content of domains and motifs, and the overall protein similarities of SbEIL 4 and 5 are ~25% compared with the other SbEILs (Figure S1).

**Figure 4:**
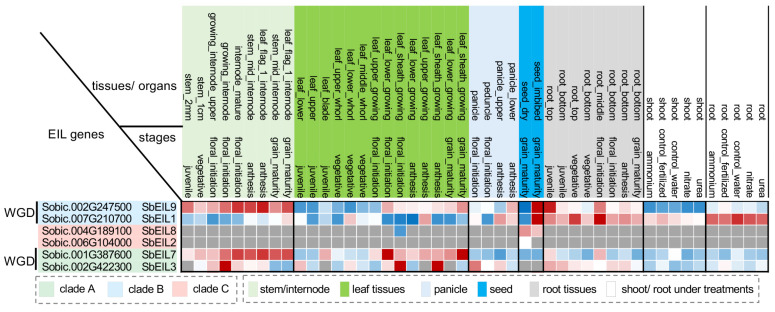
Expression profiles of SbEILs across multiple tissues and organs and developmental stages. Z-score values of the RNA-seq expression data (in FPKM) are shown in the heat map with blue and red indicating low and high expression levels, respectively. Gray means the gene was not expressed (FPKM = 0) in the corresponding tissues and stages. The expression heatmap is ordered first by the tissues/organs and then by stages. Different tissues or organs are indicated with background colors. Two pairs of WGD-derived *SbEILs* (i.e., *SbEIL1/9* and *SbEIL3/7*) are labeled, with the SbEIL phylogenetic clades indicated in green, blue, and red background colors, respectively.

We took advantage of the publicly available sorghum expression atlas to investigate the expression patterns of *SbEIL*s. Based on the expression data in the reference cultivar BTx623, *SbEIL1*, *2*, *3*, *7*, *8*, and *9* were expressed in multiple tissues and stages, whereas *SbEIL4* and *5* were not expressed (Figure 4). According to the expression data available from the Rice Gene Index, *OsEIL4* and *5* were barely expressed in the leaf, root, and panicle tissues, while they are expressed in the anther, suggesting that *SbEIL4* and *5* might also be specifically expressed in particular tissues not covered in the present sorghum expression database [40,41]. *SbEIL2* and *8* exhibited tissue-specific expression, only expressed in the seeds. By contrast, the remaining four *SbEIL*s (*SbEIL1*, *3*, *7*, and *9*) were ubiquitously expressed with clear tissue and stage specificity. For example, *SbEIL3*, *7*, and *9* were up-regulated in the leaf tissues after floral initiation. *SbEIL1* had higher expression levels in the root than in the other tissues. *SbEIL1* and *9* both showed high levels of expression during seed imbibition but were expressed at very low levels during seed maturation. Importantly, the *SbEIL* expression profiles allow us to address whether the WGD-derived *SbEIL* pairs have different expression patterns. Indeed, *SbEIL1* and *9* were differentially expressed in the stem and leaf tissues. *SbEIL9* was highly expressed in the stem and leaf after floral initiation, but *SbEIL1* had a much lower expression level. *SbEIL7* was also highly expressed in the leaf tissue after floral initiation, while the expression levels of *SbEIL3* in the corresponding tissues were lower. Collectively, these data support that the WGD-duplicated *SbEIL*s could become divergent likely because they have evolved with differentiated expression patterns, but unlikely because of the variations in domain architectures.

### 2.3. Expression Analysis Identifies SbEIL Members with Previously Unknown Involvements in Internode Maturation

**Figure 5:**
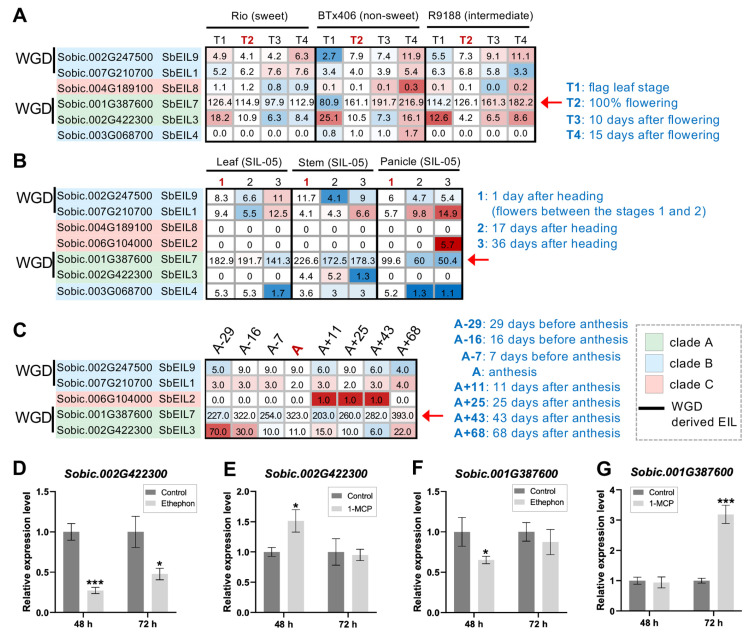
Comparative expression analysis of *SbEIL*s in the internode tissues between several sorghum cultivars. (**A**) The heat map showing the expression profiles of *SbEIL*s in three genetically related but phenotypically contrasting sorghum cultivars (sweet sorghum Rio, grain sorghum BTx406, and introgression line R9188). (**B**) The heat map showing *SbEIL*s’ expression profiles at the leaf, stem, and panicle tissues 1 day after heading (1 DAH), 17 DAHs, and 36 DAHs. (**C**) The heat map showing *SbEIL*s’ time-series expression patterns at the 10th internode from 29 days before anthesis to 68 days after anthesis (abbreviated as A-29, A-16, A-7, A, A+11, A+25, A+43, and A+68). Only expressed *SbEIL*s (averaged FPKM or RPKM values ≥ 1 per tissue and stage) are shown in the above heat maps. The colors of the heat map reflect the extent of the log2 fold change in gene expression levels relative to the flowering stage, while for Figure 5B, the gene expression fold changes were calculated to compare with those of the 1 day after heading (1 DAH). The absolute expression levels are labeled on the heat map. The flowering stages are indicated in red fonts (with “A” standing for anthesis). The red arrowhead indicates *SbEIL7* for which a further co-expression analysis was performed. Two pairs of WGD-derived *SbEIL*s (i.e., *SbEIL1/9* and *SbEIL3/7*) are labeled, with the *SbEIL* phylogenetic clades indicated in green, blue, and red background colors, respectively. (**D**–**G**) Quantitative PCR analysis of the expression levels of *SbEIL3* and *7* (Sobic.002G422300 and Sobic.001G387600, respectively) under the treatments of ethephon and 1-methylcyclopropene (1-MCP). The qPCR analysis of *SbEIL3* expression in the germinated sorghum shoot (cv. BTx623) after a 48 h or 72 h treatment of ethephon (**D**) or 1-MCP (**E**). The qPCR analysis of *SbEIL7* expression in the germinated sorghum shoot (cv. BTx623) after a 48 h or 72 h treatment of ethephon (**F**) or 1-MCP (**G**). Details about the treatments during sorghum seed germination are described in the Methods section. The qPCR analysis was performed with three biological replicates, and the statistical difference between the control and corresponding treatment was calculated with Student’s *t*-test (*, *p* < 0.05; ***, *p* < 0.005).

EILs have been known as a key component of the ethylene signaling pathway and have been involved in growth and responses to biotic and abiotic stresses in the model plant species [26,33]. Sorghum has been proposed as a research model for grass bioenergy crops with high biomass yields and the stems represent a major component of the biomass [44,45]. Thus, identifying regulators involved in sorghum stem growth, maturation, and carbohydrate allocation during the post-elongation stages is of great interest [46]. To this end, we utilized the previously reported expression data sets to understand the potential roles of *SbEIL*s in the development of sorghum stems/internodes [47,48,49,50] (Figure 5). Among the *SbEIL*s, *SbEIL2* and *8* were barely expressed in the internode. *SbEIL5* was not expressed in the internode samples, while *SbEIL4* was expressed at relatively low levels with variations between the data sets (Figure 5). In turn, the two pairs of WGD-duplicated *SbEIL*s (*SbEIL1/9* and *SbEIL3/7*) represent major *EIL* genes expressed in the sorghum internode samples. Particularly, we noticed that *SbEIL7* which is orthologous to *OsEIL6/7* (LOC_Os03g20780 and LOC_Os03g20790) was slightly down-regulated in the internode from sweet sorghum (i.e., Rio, SIL-05, and Della) but significantly up-regulated in non-sweet sorghum cultivars (BTx406 and R9188). In BTx406 internodes, the *SbEIL7* expression level was increased from 80 to 216 (in FPKM), while the *SbEIL7* expression level was up-regulated to a lesser extent in the R9188 internodes (from 114 to 182 FPKM values), correlating with the sugar-accumulating phenotypes between the cultivars Rio (high sugar contents), R9188 (intermediate sugar contents), and BTx406 (low sugar contents) (Figure 5A). *SbEIL3* was highly expressed in the internode tissue but down-regulated after flowering in both Rio, SIL-05, and Della. The distinct expression patterns and absolute levels of *SbEIL3* and *7* suggest that these two *SbEIL*s likely play a role in sorghum internodes and their functions could be different. In rice, qPCR expression analysis indicated that *OsEIL1* and *2* were the two highly expressed *OsEIL* members among the leaf, root, and panicle organs. Our RNA-seq analysis of *SbEIL*s found that *SbEIL7* (the syntenic ortholog of *OsEILs 6* and *7* [*OsEIL1* by Yang et al.], Sobic.001G387600) was the *SbEIL* gene with the highest expression level ranging from ~72 FPKM to 451 FPKM, whereas *SbEIL3* (the syntenic ortholog of *OsEIL3/OsEIL2*, Sobic.002G422300) had comparable expression levels with the other *SbEIL*s (*SbEIL1* and *9*), ranging from ~1 FPKM to ~15 FPKM. The expression levels of *SbEIL7* were higher than the other *SbEIL*s with one order of magnitude (Figure 4; Table S1). Thus, we reason that *SbEIL7* could be the major *EIL* member to regulate ethylene signaling in sorghum, while other SbEILs might have regulatory roles or functions in particular cell types not abundant in the tissues.

We employed qPCR analysis to determine whether *SbEIL3* and *7* could be involved in ethylene responses. BTx623 seeds were germinated with or without the treatments of ethylene or 1-methycyclopropene (1-MCP, an ethylene inhibitor), and the germinated shoots after 48 h or 72 h of the treatments were subject to expression analyses. Indeed, *SbEIL3* and *7* were repressed by the ethephon treatment, while they were up-regulated in the 1-MCP treatment, consistent with the proposed expression responses to ethylene signaling (Figure 5D–G).

### 2.4. SbEIL7 Is Functionally Associated with Protein Degradation during Internode Maturation

**Figure 6:**
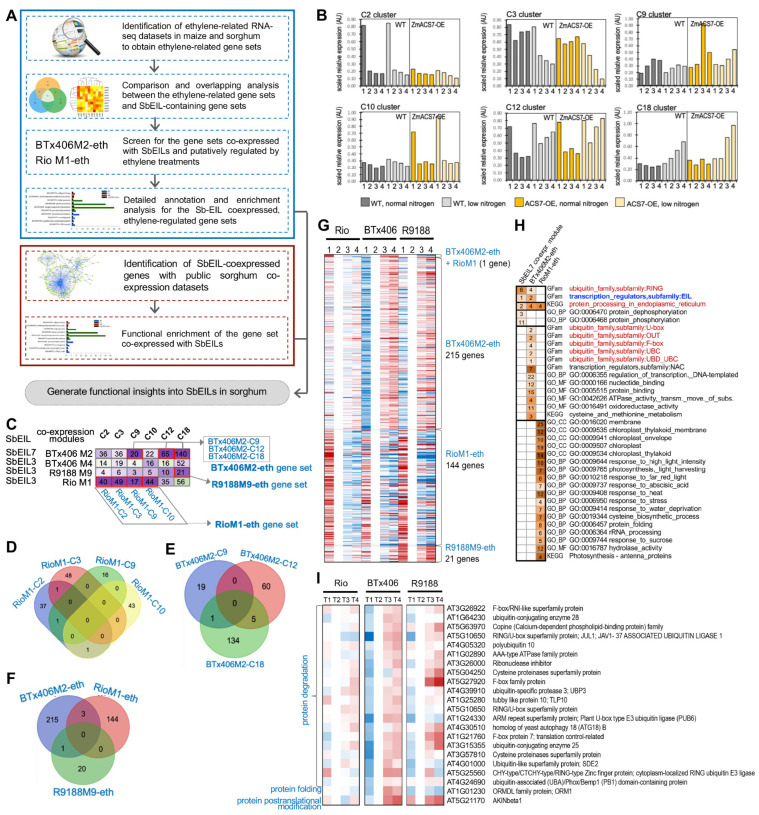
Comparison of *SbEIL* co-expressed genes with ethylene-related gene sets implicates *SbEIL*’s role in protein degradation during internode maturation stages. (**A**) The flowchart showing the process of *SbEIL* co-expression analysis and the comparison with the gene sets potentially regulated by ethylene. A literature review identifies six gene sets in maize of which their expression patterns were regulated by the ethylene signaling pathway (namely C2, C3, C9, C10, C12, and C18). The sorghum orthologs of the six gene sets were compared with *SbEIL*-containing co-expression modules (namely RioM1, BTx406M2, and R9188M9) to generate the intersection gene sets (designated as RioM1-C2, RioM1-C3, RioM1-C9, and RioM1-C10 and BTx406M2-C9, BTx406M2-C12, and BTx406M2-C18) that were significantly enriched with ethylene regulated genes (*P_hypergeometric_* < 0.05). (**B**) The expression patterns of sets of genes that are regulated by the ethylene signaling pathway (due to *ZmACS7* overexpression) under normal and low-nitrogen treatments [52]. In the bar plots, relative expression levels scaled from 0 to 1 were used. (**C**) The Venn diagram of the gene sets RioM1-C2, RioM1-C3, RioM1-C9, and RioM1-C10 showing these gene sets have only a few overlapped genes; we, therefore, combine these gene sets into one set namely RioM1-eth. (**D**) The Venn diagram of the gene sets BTx406M2-C9, BTx406M2-C12, and BTx406M2-C18 showing these gene sets have only a few overlapped genes; we, therefore, combine these gene sets into one set namely BTx406M2-eth. (**E**) The Venn diagram of the gene sets RioM1-eth, BTx406M2-eth, and R9188M9-eth. (**F**) Significant overlaps between the *SbEIL*-containing co-expression modules of sorghum internode RNA-seq data (i.e., BTx406M2, BTx406M4, RioM1, and R9188M9) and the gene sets potentially regulated by ethylene (i.e., C2, C3, C9, C10, C12, and C18) were detected (*P_hypergeometric_* < 0.05) and are shown in the heatmap. Green and purple colors reflect the log10 (*p* values) of the hypergeometric test, while purple stands for significantly over-enriched, and green means under-enriched. The significant overlaps between the gene sets were highlighted with red boxes. (**G**) The heat map showing the expression patterns of the gene sets RioM1-eth, BTx406M2-eth, and R9188M9-eth. Blue and red colors reflect the extent of log2 (fold change) relative to the anthesis stage (T2). (**H**) Functional enrichment analysis of the gene sets RioM1-eth, BTx406M2-eth, and R9188M9-eth calculated by hypergeometric tests highlights that protein degradation- and processing-related genes are enriched in the ethylene-regulated sorghum gene sets. The heatmap shows log10 (*p* values) with the numbers on the heatmap indicating the number of genes associated with the functional term. (**I**) The expression patterns of protein degradation-related genes in the internodes of Rio, BTx406, and R9188.

Our expression analysis indicated important roles of *SbEIL3* and *7* during internode development. Considering that the tandemly duplicated *OsEIL6/7* (as known as *OsEIL1*, LOC_Os03g20780) has been reported to be a major ethylene signaling component and functions in rice root growth and development, we sought to address whether *SbEIL3* and *7* could be related to ethylene signaling and the regulation of downstream genes. For this, we established a comparative transcriptome analysis scheme: (1) first, we used ethylene-related differentially expressed gene sets to analyze the *SbEIL*-containing co-expression modules; and (2) second, we used *SbEIL*-containing co-expression modules and functional enrichment analysis to gain insights into potential roles of *SbEIL3* and *7* (Figure 6A). It has been demonstrated that the gene regulatory patterns between syntenic orthologous genes in closely related species could be comparable. Maize and sorghum represent such a pair of species with a close evolutionary relationship, and a set of 15,231 syntenic orthologous gene pairs was conserved between maize subgenome 1 and sorghum, and 9553 syntenic orthologous gene pairs were conserved between the maize subgenome 2 and sorghum [35,51]. We*, therefore,* searched the literature to identify RNA-seq expression data related to ethylene signaling in maize and sorghum, allowing us to find the data set about ethylene-related transcriptome reprogramming due to the overexpression of *ZmACS7* in maize [52]. ACS7 is a well-studied ethylene biosynthetic gene in *Arabidopsis thaliana* involved in root growth and vascular meristem activity [53,54]. *ZmACS7* overexpression enhanced ethylene release and led to accelerated leaf senescence at the low-nitrogen condition [52]. The differentially expressed genes (DEGs) between the wildtype and *ZmACS7* overexpression lines (14,146 DEGs) were separated into nineteen clusters, and six gene clusters (designated as C2, C3, C9, C10, C12, and C18) revealed differential expression patterns between the WT and *ZmACS7*-OE lines under normal or low-nitrogen conditions, thereby reflecting ethylene-related transcriptome changes (Table S2). The expression patterns of these six ethylene-related clusters are shown in Figure 6B. According to the established syntenic orthologous relationship between maize and sorghum, we translated the six ethylene-related gene clusters from maize into sorghum geneIDs [35]. In the Rio/BTx406/R9188 co-expression data, clusters C9, C12, and C18 were significantly overlapped with the *SbEIL7*-containing module BTx406M2 (*P_hypergeometric_* < 0.05), leading to the gene sets potentially regulated by ethylene (namely BTx406M2-C9, BTx406M2-C12, and BTx406M2-C18) (Figure 6C). Similarly, clusters C2, C3, C9, and C10 were significantly overlapped with *SbEIL3*-containing modules RioM1, producing the four gene sets potentially regulated by ethylene (namely, RioM1-C2, RioM1-C3, RioM1-C9, and RioM1-C10). The C18 cluster also overlapped with the R9188M9 module that contains SbEIL3, yielding the overlapped gene set R9188M9-eth (“eth” stands for ethylene). Further, a comparison of the four gene sets RioM1-C2, RioM1-C3, RioM1-C9, and RioM1-C10 demonstrated that they have few overlapped genes and could be merged into one gene set (namely RioM1-eth) to represent the genes potentially regulated by ethylene signaling and co-expressed with SbEIL3 (Figure 6D). Similarly, the gene sets BTx406M2-C9, BTx406M2-C12, and BTx406M2-C18 were merged to be the BTx406M2-eth gene set (Figure 6E). Comparison between the gene sets of RioM1-eth (144 genes co-expressed with *SbEIL3*), BTx406M2-eth (215 genes co-expressed with *SbEIL7*), and the R9188M9-eth allows us to obtain a deeper understanding of ethylene-related transcriptome changes in sorghum internodes (Figure 6F; Table S3). The BTx406M2-eth gene set was significantly up-regulated after flowering in the internodes of BTx406 and R9188 but remained stable in Rio. The R9188M9-eth gene set was first down-regulated from the pre-anthesis to the anthesis stage, and then up-regulated during the post-anthesis stages in R9188, but remained relatively stable in the internodes from BTx406 and Rio. By contrast, the expression levels of the RioM1-eth gene set were dramatically decreased in Rio and R9188 after the T1 stage (Figure 6G).

To gain a deeper understanding of representative functions in the *SbEIL*-co-expressed genes, potentially ethylene-regulated gene sets, gene set enrichment analysis (GSEA) was performed (see Section 3.4 in Methods), and, interestingly, we found that these gene sets were highly enriched by protein processing- and degradation-related functions (such as “protein processing in endoplasmic reticulum”, and several gene families annotated as “ubiquitin family, RING subfamily”). Particularly, the *SbEIL7* co-expressed genes (BTx406M2-eth) include a set of ubiquitin family genes (e.g., the U-box subfamily, the F-box subfamily, and the ubiquitin-conjugating enzyme (UBC) subfamily) (Figure 6H). We further plotted the expression patterns of these genes annotated as the ubiquitin-related genes and identified their Arabidopsis best BLAST hits [34] (Figure 6I). The identified ubiquitin-related genes cover a lot of gene functions in protein degradation and protein turnover, which all exhibited up-regulated expression patterns in BTx406 and R9188 but remained relatively stable in sweet sorghum Rio [55]. In particular, the *autophagy gene 18* (*ATG18b*) was among the identified gene list, which is required for autophagy regulation during nutrient stress and senescence in Arabidopsis [56]. Additionally, the sorghum homolog of the SnRK1 subunit *AKINbeta1* (AT5G21170) showed a similar expression profile as mentioned above, which is one of the key regulatory subunits of the energy regulation kinase SnRK1 [57]. Collectively, a detailed analysis of the representative functions of *SbEIL*-co-expressed genes implies that *SbEIL7* might be involved in ethylene-mediated regulation during internode maturation stages and supports that *SbEIL7* might be functionally linked with protein processing and degradation and autophagy. 

Generally, our research work provides three interesting findings: (1) the *SbEIL* family has been expanded driven by WGD; (2) WGD-duplicated *SbEIL* pairs may become divergent because of the distinct expression profiles; and (3) co-expression analyses of sorghum developmental expression atlas and maize ethylene-related DEGs suggest that *SbEIL7* might be functionally linked with protein processing and degradation and internode maturation. The EIL transcription factors serve as a key component of ethylene signaling conserved among higher plants and the encoding gene family has been characterized genome-wide in major cereal crops, such as rice and maize [25,26]. Notably, the previous *OsEIL*s’ characterization provided detailed expression data, well supporting divergence between the WGD-duplicated *OsEIL* pairs (i.e., *OsEIL3*-*OsEIL6/7* and *OsEIL1*-*OsEIL9*) [26]. For instance, *OsEIL1* was preferentially expressed in the anther, pistil, and endosperm tissues, while *OsEIL9* was highly expressed in the leaf blade, root, panicle, and embryo tissues. *OsEIL3* was highly expressed in stems, panicles, and leaf blades, whereas *OsEIL6* had the highest expression levels in the root, followed by leaf sheath and endosperm. Unlike *SbEIL7*, the rice Nipponbare genome has a tandemly duplicated *EIL* pair (*OsEIL6* (LOC_Os03g20780) and *OsEIL7* (LOC_Os03g20790)). According to public expression data of rice, *OsEIL6* and *7* have likely become divergent as their expression profiles varied across different organs and developmental stages [26]. Even though the expression profiles across developmental stages and organs could not be directly compared between rice and sorghum, it appears that the two pairs of WGD-duplicated *EIL*s might become divergent in different ways between rice and sorghum, which deserves further detailed comparisons.

It is interesting that we discovered a potential link between SbEIL7 and protein degradation-related genes, such as several components in the ubiquitin–proteasome system (UPS, Figure 6H,I). It is noteworthy that whether SbEIL7 or ethylene signaling has a functional relationship with ubiquitin-mediated protein degradation remains to be experimentally investigated, as co-expression analysis was only employed here, which is one limitation of the present study. The UPS requires three types of enzymes, including ubiquitin-activating enzymes, ubiquitin-conjugating enzymes, and ubiquitin ligases (usually known as the E1, E2, and E3 enzymes, respectively) [58]. The ubiquitin E3 enzyme, as the UPS component with the most members, can be further divided into single-subunit subfamilies (including the HECT (homology to E6-AP C-terminus) group and the RING (really interesting new gene) group) and multi-subunit subfamilies, with the latter including the SKP1-Cullin 1-F-box (SCF) group, the VHL-ELONGIN-CUL2/5 group, the BTB-CUL3 group, the UV-damaged DNA-binding protein 1-Cullin 4 (DDB-CUL4) group, and the anaphase-promoting complex (APC) group. Among the SbEIL7-co-expressing genes related to protein degradation, we found two genes encoding E2 enzymes (homologs of AT1G64230 and AT3G15355), and several genes encoding F-box E3 enzymes (the homologs of AT3G26922, AT5G27920, and AT1G21760) and RING-type E3 enzymes (the homologs of AT5G10650; Figure 6I). The SCF complex requires both F-box proteins and RING box1 proteins, among which the F-box proteins determine the target specificity of UPS [59,60]. Particular F-box-encoding genes were identified to be co-expressed with SbEIL7 which implies that the SbEIL7-associated protein degradation might target certain proteins. Our speculation emphasizes the necessity to obtain genetic materials of sorghum in which SbEIL7 was silenced by RNAi or knocked out by the CRISPR-Cas editing technology. In addition, the treatments of ethylene and ethylene inhibitors to sorghum internodes might help validate the relationship between ethylene signaling, protein degradation, and internode maturation. The ubiquitination-mediated protein degradation process also serves as an important step to regulate ethylene signaling, as EIN3 BINDING F-BOX1/2 (EBF1/2) are the two F-box proteins specifically targeting EIN3/EIL1 and mediate EIN3/EIL1 proteolytic cleavage [12]. Our search for gene functions with the TAIR database and in the literature failed to identify any links between EBF1/2 and the Arabidopsis protein degradation-related homologs identified herein, thus leaving us to speculate that the F-box proteins here highly expressed in sorghum internodes might have targets yet to be discovered. In addition, we noted that these protein degradation-related genes were highly expressed in BTx406 and R9188, two sorghum cultivars that could not accumulate high contents of sugars (Figure 6I). Previous comparative transcriptomic studies suggest that sweet sorghum cultivars (such as Rio) have active cell metabolic status and the internodes become one of the sink organs, while in non-sweet cultivars, the internodes have inactive metabolic status and are subject to controlled senescence, and the filling seeds, but not the internodes, are the sink organ [50]. This allows us to imagine that during the late stages (e.g., 15 days after anthesis), internodes of BTx406 or R9188 may begin to become senescent, in which protein degradation to facilitate nitrogen remobilization is an important step [61]. In line with the protein degradation and senescence process, ethylene is a known phytohormone to positively regulate senescence of leaves and other organs (e.g., flowers) [62,63]. To summarize, this work leads us to the future research direction regarding the roles of ethylene signaling in internode maturation and senescence in non-sweet sorghum internodes.

## 3. Materials and Methods

### 3.1. Genome-Wide Identification and Sequence Analysis of Sorghum EILs

To identify *SbEIL* genes in a genome-wide manner, a combination of both the BLAST-based method and protein domain searching method was employed. The reference genome of sorghum cultivar BTx623 v3.1.1 was downloaded from Phytozome v13 [64], while the maize genome B73 v4 was used from the maizeGDB database [65]. The rice (*Oryza sativa* ssp. *japonica*) reference genome of Nipponbare was obtained from EnsemblPlants, and the Arabidopsis genome was from the TAIR 10.0 database [66]. For BLAST-based identification, the protein sequences of AtEILs and OsEILs were determined according to previous studies and used for BLAST [15,18,26,38]. For protein domain searching, the hidden Markov model (HMM) of the EIN3 domain (PF04873) was downloaded and used for gene identification with the Simple HMM Search function (E-value < 10^−10^) of the TBtools II package [67]. After combining the identified proteins from both methods, the SbEIL proteins were further validated by confirming whether they contain a complete EIN3 domain with the InterPro website (http://www.ebi.ac.uk/interpro/; accessed on 30 May 2024). The pI, molecular weight, instability index, and aliphatic index of SbEIL proteins were predicted with the ProtParam tool (https://web.expasy.org/protparam/; accessed on 30 May 2024). Subcellular localization was predicted with the WOLF PSORT tool (https://wolfpsort.hgc.jp; accessed by 30 May 2024).

### 3.2. Phylogenetic Analysis Sorghum EILs

The protein sequences of OsEILs, AtEILs, and ZmEILs were used for phylogenetic analysis, while all predicted protein sequences of SbEIL transcripts were used. The protein sequences were aligned with the MUSCLE method [68]. The phylogenetic tree was constructed for the EIL family proteins with the maximum-likelihood method with 1000 bootstrap replicates by using the MEGA-X software [69]. An iTOL tree was used to help with the visualization of the phylogenetic tree [70]. To interpret the phylogenetic clades from evolutionary perspectives, the information regarding the ancient whole-genome duplication of Poaceae grasses, paleo-tetraploidization of maize, and maize–sorghum comparative genomics were used based on previous studies [28,29,30,31,35].

### 3.3. Expression Analysis of Sorghum EIL

The expression profiles of *SbEIL*s were investigated from two aspects: one is the global expression across multiple tissues, organs, and developmental stages, while the other one focuses on the time-series internode expression and identifies *SbEIL* members involved in internode elongation and/or maturation.

First, the BTx623 expression atlas including several tissues/organs and stages was investigated, which includes five types of tissues and organs (i.e., leaf, stem/internode, panicle, seed, and root) as well as the shoots and roots under various nitrogen treatments (e.g., water without nitrogen, urea, nitrate, and ammonium) [34]. Developmental stages, such as juvenile, vegetative, floral initiation, anthesis, and grain maturity stages, were also included in the analysis. Second, time-series RNA-seq data of the sweet sorghum cultivar Della’s internodes were analyzed, ranging from floral induction (the A-29 stage) to post maturity (the A+43 and A+68 stages) [47]. For the Della internode RNA-seq data, “A” stands for anthesis, while the stages are named relative to the days before or after anthesis. In addition, two sets of comparative RNA-seq data were used. Rio, BTx406, and R9188 are three genetically related and phenotypically contrasting accessions. Rio is a representative sweet sorghum accumulating high contents of soluble sugars in the stem [71,72], while BTx406 is a juicy stem dwarf grain sorghum, with R9188 being the introgression line with most parts of the genetic background from Rio [49]. The expression profiles at four time points were analyzed, including the flag leaf stage, flowering, and 10 and 15 days after flowering, namely T1, T2, T3, and T4, respectively. Another comparative RNA-seq data set is the leaf, panicle, and internode tissue from the Japanese sweet sorghum SIL-05 sampled at 1 day, 17 days, and 36 days after heading [48]. In short, the Della, Rio/BTx406/R9188, and SIL-05 data sets make up an effective resource for identifying genes involved in the cessation of internode elongation, maturation, and carbohydrate metabolism from independent cultivars [50].

### 3.4. Co-Expression Networks and Functional Enrichment Analysis

Co-expression modules represent groups of genes with highly interconnected expression patterns across multiple samples and often include representative gene sets with similar biological functions [73]. Thus, co-expression networks of sorghum internode RNA-seq data serve as additional resources to further identify candidate genes related to certain biological functions in the internode. The co-expression modules of the internodes of Rio, BTx406, and R9188 have been constructed with the weighted gene expression network analysis (available as supplemental files in the corresponding reference) [49]. The co-expression modules containing *SbEIL3* and *SbEIL7* were analyzed for their enriched functions with gene ontology (GO) annotations. Functional enrichment analysis was performed with the gene set enrichment analysis (GSEA) function of the SorghumFDB (with the hypergeometric test and significance determined by using the Hochberg-adjusted FDR < 0.05) [74,75].

In addition, to identify the sorghum genes potentially regulated by the ethylene signaling pathway in the internode, RNA-seq data sets related to ethylene signaling were searched for in the literature. The ethylene-regulated gene sets in maize were obtained by comparing *ZmACS7* overexpression lines and their corresponding wildtype, and further transferred to sorghum geneIDs by using the established gene orthologous relationships between sorghum and maize considering the fact that the split of the progenitors of sorghum and maize was ~12 million years ago (MYAs) and a large portion of the orthologs could be conserved and comparable between sorghum and maize [35,52,76].

### 3.5. Quantitative Polymerase Chain Reaction (qPCR) Analysis

Seeds of sorghum cultivar BTx623 were germinated in Petri dishes with the treatment of ethephon (50 μM), 200 mg/L 1-MCP (ethylene inhibitor), or water (control), respectively. The germinated shoots were sampled at 48 h or 72 h after the treatment for RNA extraction, with the experiments carried out in triplicates. Total RNA was extracted by using an RNA Extraction Kit (Zomanbio, Beijing, China) and reversely transcribed into cDNA for qRT-PCR as described previously [50]. qPCR reactions were conducted by using SYBR Green Master Mix with the CFX96 real-time System (Bio-RAD, Hercules, CA, USA). Relative expression levels were calculated using the ΔΔCT method with the sorghum ubiquitin gene used as the internal reference for qPCR analysis [51]. For the quantification of Sobic.002G422300, the forward primer is “GAGACGATGGCGATGACCTTCC” (from 5′ to 3′), while the reverse primer is “GGTCACTGATCTGCTGCTGCTG”. For the quantification of Sobic.001G387600, the forward and reverse primers are “TCCAAGTGCCTCCAGGACAAGA” (from 5′ to 3′) and “GCGATGCCACCAGTAGATGCT” (from 5′ to 3′), respectively.

## 4. Conclusions

In conclusion, our work identified eight SbEIL-encoding genes in sorghum and revealed their evolution trajectory, highlighting the values of combining genome-wide analysis and duplication information and the WGD-driven expansion of *SbEIL*s. Comprehensive expression analyses demonstrated the divergence of the potential functions between WGD-duplicated *SbEIL* pairs could be due to differentiated expression profiles. Moreover, we characterized *SbEIL3* and *7* to be the EIL members involved in internode development and maturation. Specifically, *SbEIL7*, the syntenic ortholog of duplicated *OsEIL6/7* (the master transcription factors for the ethylene signaling in rice), might be linked to biological functions during internode maturation, such as protein processing and degradation. These results lay a foundation for functional studies for ethylene signaling-mediated gene regulation and deepen our understanding of the physiological and molecular differences in internode development between sweet and grain sorghum cultivars.

## Figures and Tables

**Table 1:** The information of the *SbEIL* family and the encoded proteins.

Gene Name	Gene ID	Phylo.	Gene Location	Protein Length (AA)	Molecular Weight (Da)	pI	Instability Index	Aliphatic Index	Predicted Subcellular Localization	N-Terminal Region	Five α-Helix Regions	BD Regions	Proline-Rich Region
SbEIL3	Sobic.002G422300	A	Chr02:76992033-76994387	594	63,959.14	6.05	55.01	64.41	nucleus	P	P	P	P
SbEIL7	Sobic.001G387600	A	Chr01:67472693-67476803	643	71,826.48	5.02	55.13	59.94	nucleus	P	P	P	P
SbEIL1	Sobic.007G210700	B	Chr07:63959262-63963026	618	68,185.64	5.72	39.19	72.3	nucleus	P	P	P	P
SbEIL9	Sobic.002G247500	B	Chr02:63544987-63550449	609	68,172.73	5.84	53.81	73.15	nucleus	P	P	P	P
SbEIL4	Sobic.003G068700	B	Chr03:5809983-5814702	322	33,714.91	5.98	43.85	80.31	nucleus	X	2, 3	X	P
SbEIL5	Sobic.002G078850	B	Chr02:8204439-8206181	511	52,199.57	5.03	54.41	77.28	nucleus	P	2, 3	X	P
SbEIL2	Sobic.006G104000	C	Chr06:47392162-47394762	470	51,819.71	5.2	52.62	64	nucleus	P	P	P	P
SbEIL8	Sobic.004G189100	C	Chr04:54101320-54103770	478	52,693	4.94	59.52	70.63	nucleus	P	P	P	P

Note: For gene locations, the chromosome number and start and end position of the gene are provided; for the predicted subcellular localization, the information is predicted by the WOLFPSORT website and provided (https://wolfpsort.hgc.jp/aboutWoLF_PSORT.html.en; accessed by 31 July 2024). For the N-terminal region, the first approximately 100 amino acid residues including the BD I and BD II regions were compared; for the five α-helix regions important for the EIL protein structure, “P” means it contains all of the five α-helix regions, while “2, 3” means it only contains the second and third α-helix regions. The column “Phylo.” shows the phylogenetic clades each SbEIL belongs to.

## Data Availability

The data presented in this study are available in the article and the Supplementary Materials. For further inquiries, you can contact the corresponding author directly.

## Supplementary Materials

The following supporting information can be downloaded at https://www.mdpi.com/article/10.3390/plants13182615/s1: Figure S1: The similarity matrix between SbEIL and OsEIL proteins. The protein similarity matrix reflecting the sequence identity between each pair of OsEILs and SbEILs was calculated with the Clustal-Omega program on the EMBL website. OsEILs and SbEILs were aligned with the Clustal-Omega method. The sequence similarity percentages (ranging from 0% to 100%) are shown in the heatmap with blue and red indicating the lowest and highest sequence similarity percentages, respectively. OsEILs and SbEILs belonging to the same phylogenetic clades (Figure 1) are indicated with black boxes, and the gene names of OsEILs are given in green backgrounds; Table S1: Expression profiles of the *SbEILs* in the BTx623 expression atlas (expression values are given as FPKM) (provided in a separate EXCEL file); Table S2: The geneIDs translated from maize ethylene-regulated gene sets (i.e., C2, C3, C9, C10, C12, and C18) (provided in a separate EXCEL file); Table S3. The gene sets overlapped between *SbEIL-*containing co-expression modules of Rio/BTx406/R9188 and those potentially regulated by ethylene (C2, C3, C9, C10, C12, and C18), yielding an overlap (provided in a separate EXCEL file).
