# Supplementary material for: Characterization and Transcriptomic Analysis of Sorghum EIN/EIL Family and Identification of Their Roles in Internode Maturation
Source: Plants (Basel). 2024 Sep 19;13(18):2615. doi: 10.3390/plants13182615 (PMC11435218; doi:10.3390/plants13182615)
Supplement: Supplementary file 1 [file plants-13-02615-s001.zip › Mint_SbEIL_supplV1_20240804.pdf]

Supplementary Information for

Characterization and Transcriptomic Analysis of Sorghum EIN/EIL Family and Identification of Their Roles in Internode Maturation

Min Tu [1,*,†], Yuqing Hua [1,†], Ti Shao [2], Siyu Zhang [1], Zihan Xiang [3], Manting Yu [3], Guoli Wang [2], Zhuang Li [1], Yun He [1], Lin Yang [1] and Yin Li [2,*]

1 Hubei Technical Engineering Research Center for Chemical Utilization and Engineering Development of Ag-ricultural and Byproduct Resources, School of Chemical and Environmental Engineering, Wuhan Polytechnic University, Wuhan 430023, China

2 The Genetic Engineering International Cooperation Base of Chinese Ministry of Science and Technology, Key Laboratory of Molecular Biophysics of Chinese Ministry of Education, College of Life Science and Technology, Huazhong University of Science and Technology, Wuhan 430074, China

3 School of Mathematics and Computer Science, Wuhan Polytechnic University, Wuhan 430023, China

* Correspondence: 12739@whpu.edu.cn (M.T.); yinli2021@hust.edu.cn (Y.L.)

† These authors contributed equally to this work.

	SbEIL9_Sobic.002G247500	OsEIL9_LOC_Os09g31400	SbEIL1_Sobic.007G210700	OsEIL1_LOC_Os08g39830	OsEIL4_LOC_Os07g17160	SbEIL4_Sobic.003G068700	OsEIL5_LOC_Os07g12210	SbEIL5_Sobic.002G078850	SbEIL2_Sobic.006G104000	OsEIL2_LOC_Os04g38400	SbEIL8_Sobic.004G189100	OsEIL8_LOC_Os02g36510	SbEIL3_Sobic.002G422300	OsEIL3_LOC_Os07g48630	SbEIL7_Sobic.001G387600	OsEIL7_LOC_Os03g20790	OsEIL6_LOC_Os03g20780
SbEIL9_Sobic.002G247500	100	77	58	58	18	24	19	23	36	37	37	35	43	44	43	43	43
OsEIL9_LOC_Os09g31400	77	100	59	59	19	24	20	24	36	37	37	37	42	42	42	43	43
SbEIL1_Sobic.007G210700	58	59	100	62	19	21	21	23	37	38	38	37	41	44	44	43	43
OsEIL1_LOC_Os08g39830	58	59	62	100	19	24	20	24	34	36	36	35	41	41	44	43	43
OsEIL4_LOC_Os07g17160	18	19	19	19	100	25	25	28	22	21	20	19	22	25	25	26	26
SbEIL4_Sobic.003G068700	24	24	21	24	25	100	34	72	26	26	26	27	24	25	25	25	25
OsEIL5_LOC_Os07g12210	19	20	21	20	25	34	100	34	18	20	20	25	21	24	21	21	21
SbEIL5_Sobic.002G078850	23	24	23	24	28	72	34	100	25	26	23	26	22	23	23	24	24
SbEIL2_Sobic.006G104000	36	36	37	34	22	26	18	25	100	68	54	55	36	37	36	36	36
OsEIL2_LOC_Os04g38400	37	37	38	36	21	26	20	26	68	100	59	61	41	42	43	43	43
SbEIL8_Sobic.004G189100	37	37	38	36	20	26	20	23	54	59	100	65	40	40	41	41	41
OsEIL8_LOC_Os02g36510	35	37	37	35	19	27	25	26	55	61	65	100	39	40	41	42	42
SbEIL3_Sobic.002G422300	43	42	41	41	22	24	21	22	36	41	40	39	100	67	58	59	59
OsEIL3_LOC_Os07g48630	44	42	44	41	25	25	24	23	37	42	40	40	67	100	66	67	66
SbEIL7_Sobic.001G387600	43	42	44	44	25	25	21	23	36	43	41	41	58	66	100	85	85
OsEIL7_LOC_Os03g20790	43	43	43	43	26	25	21	24	36	43	41	42	59	67	85	100	99
OsEIL6_LOC_Os03g20780	43	43	43	43	26	25	21	24	36	43	41	42	59	66	85	99	100

Supplementary Figure S1. The similarity matrix between SbEIL and OsEIL proteins. The protein similarity matrix reflecting the sequence identity between each pair of OsEILs and SbEILs were calculated with the Clustal-Omega program in the EMBL website. OsEILs and SbEILs were aligned with the Clustal-Omega method. The sequence similarity percentages (ranging from 0% to 100%) are shown in the heatmap with blue and red indicating the lowest and highest sequence similarity percentage, respectively. OsEILs and SbEILs belong to the same phylogenetic clades (Figure 1) are indicated with black boxes, and the gene names of OsEILs are given in green backgrounds.

Supplementary Table S1. Expression profiles of the *SbEIL*s in the BTx623 expression atlas. (expression values are given as FPKM) (provided in a separate EXCEL file)

Supplementary Table S2. The geneIDs translated from maize ethylene regulated gene sets (i.e., C2, C3, C9, C10, C12, and C18). (provided in a separate EXCEL file)

Supplementary Table S3. The gene sets overlapped between *SbEIL*-containing co-expression modules of Rio/BTx406/R9188 and those potentially regulated by ethylene (C2, C3, C9, C10, C12, and C18), yielding the overlapped. (provided in a separate EXCEL file)
